# A massive pulmonary arteriovenous fistula complicated with coronary atherosclerotic heart disease treated by interventional therapy: a case report

**DOI:** 10.1186/s13019-024-02866-w

**Published:** 2024-06-27

**Authors:** Jingwen Guo, Hongyong Wang, Mingming Zhang

**Affiliations:** 1 Department of Cardiology, Daping Hospital, The Third Military Medical University (Army Medical University), No. 10 Yangtze River Branch Road, Yuzhong District, Chongqing, 400042 P.R. China; 2 Chongqing Key Laboratory for Hypertension Research, Chongqing Cardiovascular Clinical Research Center, Chongqing Institute of Cardiology, Chongqing, 400010 China

**Keywords:** Pulmonary arteriovenous fistula, Coronary atherosclerotic heart disease, Interventional therapy

## Abstract

**Background:**

Pulmonary arteriovenous fistula (PAVF) is a rare disease, and its symptoms lack specificity. For patients with coronary heart disease(CHD), hypertension and other common cardiovascular diseases, PAVF is easy to be ignored. We presented a case of massive PAVF complicated with coronary atherosclerotic heart disease by interventional treatment to improve the understanding of this complex disease.

**Case presentation:**

A 77-year-old female patient was admitted to the hospital due to chest tightness and shortness of breath following activities, which was diagnosed with CHD and hypoxemia in other hospitals. Coronary angiography showed that the patient had severe stenosis of coronary artery while pulmonary vascular DSA showing the patient had PAVF. After interventional therapy of both coronary artery and PAVF, the patient's symptoms were significantly improved.

**Conclusion:**

We presented a case of massive PAVF complicated with CHD by interventional treatment. For patients with unexplained hypoxemia and symptoms similar with CHD, the possibility of PAVF often leads to oversight, and various auxiliary examinations should be improved to avoid missed diagnosis. And intervention treatment should be carried out to improve the prognosis of patients as much as possible.

## Background

Pulmonary arteriovenous fistula (PAVF) is a rare type of cyanotic pulmonary vascular malformation with right-to-left shunt which specific pathogenesis is still unclear [1]. Hereditary hemorrhagic telangiectasia is the most common cause of PAVF, and acquired factors such as trauma, surgery, and cirrhosis could also cause PAVF. PAVF patients mainly present with hypoxemia, chronic heart failure, abnormal cerebral infarction, and brain abscess, symptoms lacking specificity and often leading to oversight [2]. Coronary atherosclerotic heart disease (CHD) is currently the prevailing chronic cardiovascular disease in China, with high incidence and diagnosis rates. The principal manifestation of the syndrome is chest tightness or chest pain after the activity [3]. Percutaneous coronary intervention (PCI) is the common treatment for coronary atherosclerotic heart disease. This paper presents a case of massive pulmonary arteriovenous fistula complicated with coronary atherosclerotic heart disease by interventional treatment to improve the understanding of this complex disease.

## Case presentation

**Fig. 1:**
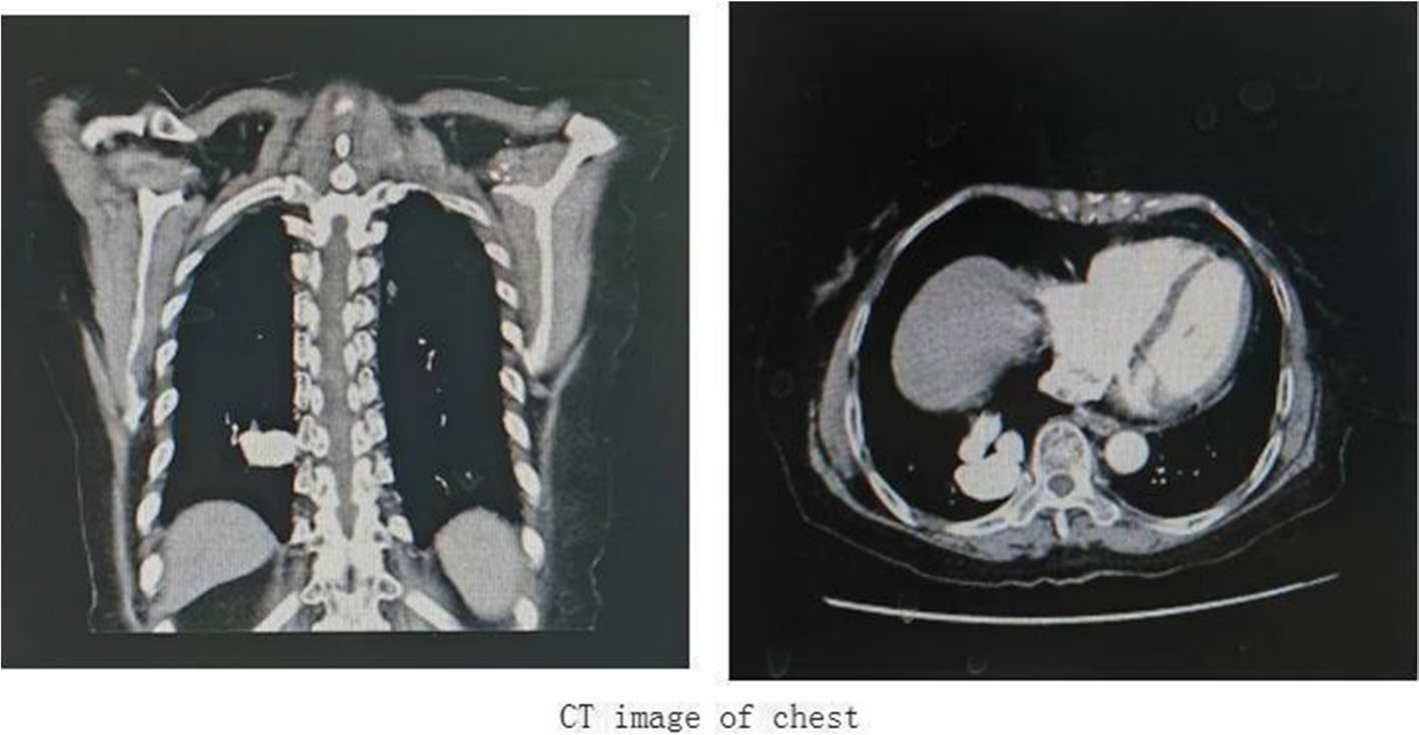
CT image of chest

A 77-year-old female patient was admitted to the hospital on November 4, 2020, due to chest tightness and shortness of breath following activities for 5 years, with exacerbation over the past six months. The patient had frequently visited other hospitals due to chest tightness and shortness of breath post-exercise, previously diagnosed with coronary heart disease and hypoxemia. Six months before admission, the patient's exercise tolerance was significantly decreased, accompanied by paroxysmal dyspnea at night. The pulse rate was 104 beats per minute, respiratory rate was 20 breaths per minute, and blood pressure measured 105/62 mmHg (1 mmHg = 0.133 kPa) at admission. The patient was clear in consciousness and had steady breathing. There were none positive signs in her cardiopulmonary physical exam. Additionally, there was moderate pitting edema in both legs. The patient denied hypertension, diabetes, hepatitis and tuberculosis of her medical history. She had no history of smoking or alcohol consumption. There were no markedly abnormal findings in her venous blood tests including markers of myocardial damage, NTproBNP, d-dimer assay and blood routine examination. Preoperative blood gas analysis showed hypoxemia with a PaO_2_ of 44mmHg and S0_2_ of 88% without oxygen. The electrocardiogram showed the patient had sinus rhythm, anomalous Q wave in her II, III and aVF leads, clockwise rotation and ST-T changes.The transthoracic echocardiography showed mild impairment of left ventricle diastolic function. The chest CT scan showed right inferior pulmonary arterioveneus malformations and multiple patchy chronic inflammatory pulmonary nodules (Fig. 1).

**Fig. 2:**
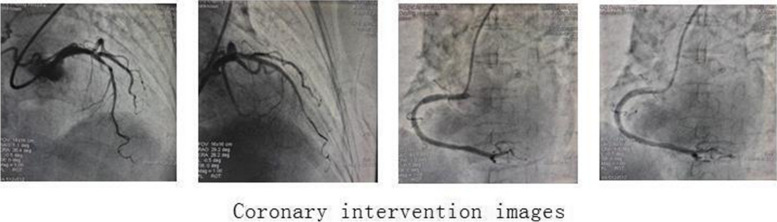
Coronary intervention images

**Fig. 3:**
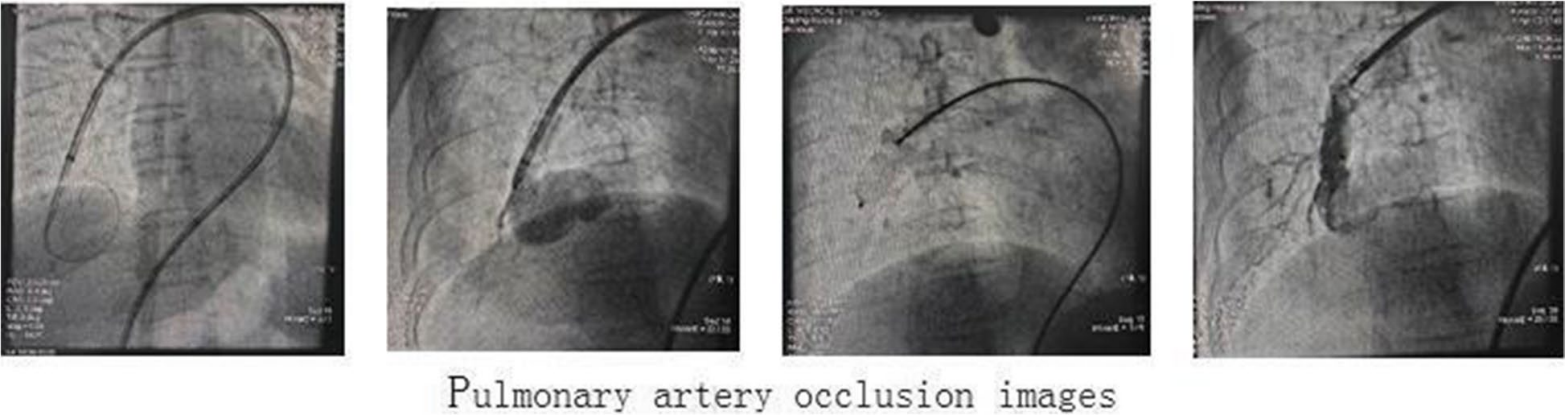
Pulmonary artery occlusion images

The symptoms of chest tightness and shortness of breath were obvious following exercise, which considered to be related to coronary atherosclerotic heart disease and PAVF. After communicating with the patient and her family, coronary angiography and pulmonary angiography were performed under local anesthesia in 6th November,2020. The results showed that the right coronary artery was dominant; the left main coronary artery was normal, and the proximal and middle segment of left anterior descending coronary artery stenosis was about 40–80%, TIMI grade 3; the middle segment stenosis of circumflex artery was about 20–30%, TIMI grade 3; the proximal and middle segment of the right coronary artery stenosis was about 40–70%, TIMI grade 3 (Fig. 2). The pulmonary angiography showed a large fistula between the right pulmonary artery and the right inferior pulmonary vein (Fig. 3). One stent was placed in the left anterior descending artery and one in the right coronary artery under local anesthesia in 6th November,2020. The patient underwent PAVF transcatheter closure under local anesthesia in 11th November,2020. During the operation, a PAVF was found in the main trunk of the right pulmonary artery. The narrowest point was about 10mm, and a tumor with a diameter of about 30mm was seen in the distal part of the PAVF. Some branches in the proximal and middle segment of the pulmonary artery fistula provided the blood supply for the right lung, and the distal segment refluxed to the left atrium. The occluder device delivery system (10F) was delivered into the right pulmonary artery fistula through the right pulmonary artery along the guide wire, and the distal end was located at the narrowest point of the right pulmonary artery fistula. The Shenzhen Xianjian patent ductus arteriosus occluder (XJFD1618) was sent to the right pulmonary artery fistula, releasing to the narrowest point of the right pulmonary artery fistula on the umbrella surface, and the delivery sheath was withdrawn to the narrowest point of the right pulmonary artery fistula on the umbrella surface.The right pulmonary arteriovenous fistula was basically blocked by the occlude, the shunt was significantly reduced, and only small amount of shunt remained. The patient's blood oxygen saturation increased rapidly from 84 to 91% without oxygen. The patient had a small amount of hemoptysis after the operation. Postoperative blood gas analysis showed that oxygenation had improved with a PaO_2_ of 59 mmHg and S0_2_ of 93% without oxygen. After operation, aspirin, clopidogrel bisulfate and atorvastatin were given. At an outpatient visit 1 month after operation, chest tightness and shortness of breath had improved and no recurrence. The quality of life was significantly improved.

## Discussion and conclusions

PAVF refers to the condition where the pulmonary artery and pulmonary vein communicate directly through enlarged tortuous blood vessels or hemangioma, resulting in a low incidence. PAVF can be categorized into three types: single, complex, and diffuse. More than 80% of PAVF patients belong to the single type, characterized by a connection between one artery and one vein without division of the cystic tumor. The complex type refers that the lesion is not limited to one artery and one vein, and the cystic tumor is usually separated. The diffuse type refers to the presence of extensive small pulmonary arteriovenous fistulas without cystic tumor formation [4, 5]. The severity of PAVF is directly related to fistula size. A single fistula less than 2cm in diameter usually does not cause symptoms. When the right-to-left shunt volume increases with the enlargement of the fistula, the symptoms such as dyspnea and cyanosis may gradually appear, and the patient's exercise tolerance gradually decreases. About 25% of patients also experience neurological symptoms such as cerebral embolism, transient ischemic attack, migraine, and brain abscess. Pulmonary artery CTA is the preferred diagnostic method for PAVF, providing an overview of the lesion and measuring the diameter of affected vessels. Pulmonary vascular DSA is the gold standard for the diagnosis of PAVF, which can observe the fistulas, feeding arteries and draining veins of PAVF.

After diagnosis, most PAVF patients require treatment. The mortality rate of untreated PAVF patients could be as high as 50%, whereas it could be reduced to 3% after treatment [6]. Current treatments for PAVF include surgery and interventional therapy. The recurrence rate of surgical treatment is low, but it is limited to the treatment of single lesion, and the trauma is large. Interventional therapy could retain more lung tissue and has less trauma, but it is not suitable for more lesions and diffuse lesions. Interventional therapy achieved its first success in treating PAVF in 1978. With the continuous improvement of surgical techniques and interventional materials, interventional treatment of PAVF has been proved to be safe and effective, and has become the first choice in clinical practice [7–10]. Interventional therapy for PAVF is mainly divided into coil occlusion and occluder occlusion. For PAVF patients with feeding artery diameter < 5 mm, coil closure is recommended. For PAVF with the diameter of feeding artery ≥ 5 mm, the use of coil will increase the risk of its detachment and displacement or even ectopic embolism, and the use of occluder is more effective and safer [11]. At present, the main conservative treatment of diffuse PAVF is improving the symptoms of hypoxia. In this case, the the narrowest diameter of the feeding artery was about 10mm through pulmonary vascular DSA. Therefore the Shenzhen Xianjiang patent ductus arteriosus occluder (XJFD1618) was used for occlusion. After occlusion, the shunt was significantly reduced and the patient's blood oxygen saturation increased rapidly. In the end, the patient's symptoms were significantly improved.

The patient's primary clinical manifestations were chest tightness and shortness of breath following activities, consistent with common symptoms of CHD. Coronary angiography showed that the patient had severe stenosis of the anterior descending artery and the right coronary artery which were consistent with the symptoms, and interventional treatment was given. However, the patient was also complicated with type I respiratory failure, and pulmonary artery CTA showed arteriovenous malformation in the right lower lobe of the lung, which was considered to have PAVF. Pulmonary vascular DSA was performed to confirm the diagnosis of right PAVF, and the patient was treated with occlusion. After the operation, the patient's hypoxemia and symptoms were significantly improved.

PAVF is a rare disease, and its symptoms lack specificity. For elderly patients with CHD, hypertension and other common cardiovascular diseases, PAVF often leads to oversight. Therefore, in patients with unexplained hypoxemia, considering the possibility of PAVF is crucial, necessitating comprehensive auxiliary examinations to prevent missed diagnosis. On this basis, intervention treatment should be carried out to improve the prognosis of patients as much as possible.

## Data Availability

No datasets were generated or analysed during the current study.

## Abbreviations

PAVF: Pulmonary arteriovenous fistula
CTA: Computed tomography angiography
CHD: Coronary atherosclerotic heart disease
DSA: Digital subtraction angiography
PCI: Percutaneous coronary intervention
